# Natural Categorization: Electrophysiological Responses to Viewing Natural Versus Built Environments

**DOI:** 10.3389/fpsyg.2020.00990

**Published:** 2020-06-10

**Authors:** Salif Mahamane, Nick Wan, Alexis Porter, Allison S. Hancock, Justin Campbell, Thomas E. Lyon, Kerry E. Jordan

**Affiliations:** ^1^Department of Behavioral & Social Sciences, Western Colorado University, Gunnison, CO, United States; ^2^Department of Psychology, Utah State University, Logan, UT, United States; ^3^Department of Psychology, Northwestern University, Evanston, IL, United States; ^4^MD-PhD Program, School of Medicine, The University of Utah, Salt Lake City, UT, United States

**Keywords:** environment, electrophysiology, LPP, ERP, natural, implicit categorization

## Abstract

Environments are unique in terms of structural composition and evoked human experience. Previous studies suggest that natural compared to built environments may increase positive emotions. Humans in natural environments also demonstrate greater performance on attention-based tasks. Few studies have investigated cortical mechanisms underlying these phenomena or probed these differences from a neural perspective. Using a temporally sensitive electrophysiological approach, we employ an event-related, implicit passive viewing task to demonstrate that in humans, a greater late positive potential (LPP) occurs with exposure to built than natural environments, resulting in a faster return of activation to pre-stimulus baseline levels when viewing natural environments. Our research thus provides new evidence suggesting natural environments are perceived differently from built environments, converging with previous behavioral findings and theoretical assumptions from environmental psychology.

## Introduction

Natural environments can increase positive emotions (Roe et al., 2013) and creativity (Atchley et al., 2012), decrease stress (Ulrich, 1984; Mayer et al., 2009; MacKerron and Mourato, 2013; Hartig et al., 2014; Kuo, 2015; Von Lindern et al., 2017; Mennis et al., 2018), decrease impulsive decision-making (Berry et al., 2014; Berry et al., 2015), and are preferred over built images by adults (Kaplan et al., 1972; Meidenbauer et al., 2019). Natural environments can also improve working memory (Triguero-Mas et al., 2015) and attention (Hartig et al., 1991; Tennessen and Cimprich, 1995; Wells, 2000; Kuo and Faber Taylor, 2004; Berto, 2005; Berman et al., 2008; Mayer et al., 2009; Debener et al., 2012; Hartmann et al., 2013; Lee et al., 2015; Dadvand et al., 2017; Torquati et al., 2017; Ulset et al., 2017; Van Hedger et al., 2018; Stevenson et al., 2019).

Attention is often required when engaging in a behavior (i.e., maintaining attention to an ongoing task). If a person continues to engage in the behavior, their perceived cost in attentional resources is likely outweighed by the potential reward gained from continuing the behavior. However, with time on task, a person’s likelihood of continuing the behavior decreases as attentional resources, which are finite, deplete. This depletion is experienced as mental fatigue (Kaplan, 1995). The fatigability of attention can thus be conceptualized as a cost-benefit analysis (Boksem and Tops, 2008), and mental fatigue can be understood as the point at which the perceived attentional costs of continuing a behavior are greater than the potential reward gained from continuing that behavior. Maintenance of attention to engage in a behavior can be guided by either top-down (endogenous and goal-driven) or bottom-up (exogenous and stimulus-driven) processes that support cognitive control (Posner and Petersen, 1990). Interacting with natural environments, such as taking a walk in the park, can improve performance on tasks designed to measure these systems of attention, especially endogenous attention (Ohly et al., 2016). Fewer cognitive resources may be needed to reach the same level of attentional performance when tested outdoors vs. indoors (Debener et al., 2012; Torquati et al., 2017). Such evidence suggests that interactions between attention and environment play a role in behavioral performance, specifically with respect to the onset of mental fatigue. Yet, there is little research to explain underlying mechanisms or physiological correlates of this relationship (Berman et al., 2019). It is important to understand biological underpinnings to this potentially transformative, yet low-cost and non-invasive, avenue for bolstering mental health and well being (Schertz and Berman, 2019).

Attention Restoration Theory (ART; Kaplan, 1989) largely drives this research. ART posits that certain environments better replenish attentional resources from a depleted state, based on five psycho-environmental characteristics: fascination, extent, coherence, being away, and compatibility. Fascination is an environment’s ability to capture exogenous attention. Extent is the degree to which the environment is of large enough scope to keep exogenous attention engaged for more than a brief moment. Coherence, as defined within ART, is the degree to which the scene makes sense as a whole. Being away represents the conceptual removal of the potentially restorative environment from the fatiguing environment. Finally, compatibility represents characteristics of the potentially restorative environment that would facilitate a person’s goals for restoration. Thus, the first three characteristics are responsible for an environment’s ability to capture and hold exogenous attention, whereas the last two conceptually distinguish environments in which mental fatigue occurs (being away) and align with a person’s restorative goals (compatibility; Kaplan, 1989, 1995; Kaplan and Berman, 2010). The theory posits that when finite resources for endogenous attention are depleted, the cognitive mechanisms for inhibiting distractions suffer. In this state of mental fatigue, environments that easily engage exogenous attention effectively allow these inhibitory mechanisms to rest and attentional resources to replenish; this is defined as attention restoration (Kaplan, 1989). Previous research has shown natural environments, when rated along these five characteristics, to be more restorative than built environments (Berto, 2005, 2007; Berman et al., 2008; Lee et al., 2015).

Studies driven by this theory concerning human responses to natural and built environments have primarily examined behavioral responses (e.g., Kaplan, 1989, 1995; Berto, 2005, 2007; Berman et al., 2008; Berto et al., 2008, 2010). Fewer studies examine how exposure to natural and built environments correlates with brain activity. Aspinall et al. (2013) conducted an EEG spectral analysis of how humans perceive walks through natural environments versus walks through built environments. Their data suggest general neural differences correlated with natural environments versus built environments, but do not pinpoint specific neural mechanisms. Another study reported cortisol differences correlated with exposure to natural versus built environments, postulating that these differences arise due to differences in stress (Roe et al., 2013). To our knowledge, though, no study has used the temporal sensitivity of EEG to investigate potential electrophysiological differences correlated with viewing images of natural versus built environments. It is possible that temporal differences measured by EEG, via event-related potentials (ERP’s), can help elucidate physiological differences in humans while viewing natural versus built environments. Employing such methodology also enables the use of implicit measures.

In the present study, we specifically focus on the p3 and late positive potential (LPP) ERP components. The p3 correlates with detection of categorical differences: greater activation is found when a rare stimulus (the ‘target,’ i.e., not a member of a category commonly experienced within the experimental paradigm) is shown (Sutton et al., 1965; Rozenkrants and Polich, 2008). For example, p3 amplitude correlates with detection of changes in category of facial features (Azizian et al., 2006). Here, we use a set of diverse stimuli to represent distinct natural versus built environmental categories; stimuli within each category were determined prior to our electrophysiological experiment by a separate sample of adults who completed an explicit, binary environmental categorization task. Then, p3 activation to these two environmental categories was recorded while participants passively viewed a high frequency of multiple non-target stimuli representing one category (e.g., natural environments) and a low frequency of target stimuli representing the opposite category (e.g., built environments). Greater p3 amplitude for target stimuli, regardless of stimulus category (natural vs. built), is hypothesized. Such a finding from this passive viewing ‘oddball’ paradigm would provide electrophysiological support for implicit categorization of environmental scenes as natural versus built.

The LPP ERP component is examined most often with respect to stimulus valence (Bradley et al., 2007; MacNamara et al., 2009, 2011) or perceived pleasantness (Hajcak and Olvet, 2008). When a given stimulus (such as a building or landscape) is perceived as pleasant or unpleasant, rather than neutral, the LPP shows a slower recovery time to baseline levels of activation, which can be quantified by greater positive mean amplitude than is observed for neutral stimuli. Negative stimuli have greater positive amplitude than pleasant stimuli, while neutral stimuli show more baseline amplitude. Simultaneous recordings of EEG and fMRI taken while participants viewed affective pictures have shown LPP and blood oxygen level-dependent (BOLD) frontal activity to indicate contributions to perceptual categorization and emotional processing (Liu et al., 2012).

Previous behavioral research shows that natural environments correlate with a decrease in negative affect and are preferred over built environments (Kahn and Kellert, 2002; Berto, 2005, 2007; Berto et al., 2008, 2010; Falk and Balling, 2010; Aspinall et al., 2013). Previous data from EEG spectral analyses also suggest that natural environments hold greater positive valence than built environments (Aspinall et al., 2013; Roe et al., 2013), but LPP was not measured in these studies. We ask in the current study using the passive oddball viewing paradigm whether natural environments not only correlate with behavioral responses indicating preference, but also electrophysiological evidence in the form of LPP differences as well.

In sum, the current study employs a passive viewing oddball task in which stimuli from each of two environmental categories occur, with one category appearing at a high frequency and the other appearing at a low frequency. We examine the p3 and LPP ERP components correlated with viewing the stimuli. Hypotheses for this study are twofold: first, if natural versus built environments are implicitly perceived as being from distinct visual categories, a greater p3 should occur after viewing a target. Second, if natural environments are perceived as more pleasant than built environments, there may be a faster LPP recovery time after viewing non-target blocks of natural environments than after viewing non-target blocks of built environments. Either such result would identify electrophysiological markers signifying that humans implicitly categorize environments along dimensions of naturalness and/or pleasantness, and move closer to elucidating potential mechanisms and neural correlates underlying benefits of human exposure to natural environments.

## Materials and Methods

### Participants

All participants signed IRB-approved consent forms. Students who participated received course credit toward their undergraduate psychology course work.

#### Categorization Sample

Fifty-one adults (21 Females; 18–38 years old; *M* = 23.58 years old) participated in a binary stimulus categorization task. Participants provided their gender, handedness, and age.

#### Rating Sample

Thirty-seven adults (21 Females, 1 unreported; 18–32 years old; *M* = 21.22 years old), different from those in the categorization sample, rated scenic images on an adapted version of the Perceived Restorative Scale (PRS) called the PRS short version (Berto, 2005; for original see Korpela and Hartig, 1996) and a preference item, which will be described below. Participants provided their gender, handedness, and age.

#### ERP Sample

Seventy-four right-handed adults (30 Females; 18–40 years old; *M* = 21.8 years old), different from those in each of the categorization and ratings samples, participated in a passive viewing task with stimuli assessed by the aforementioned categorization and rating tasks. Handedness was measured using the Edinburgh Handedness Inventory - Short Form (Veale, 2014). Data from 14 participants were rejected due to excessive noise in the signal (2), corrupted dataset (5), or EEG-to-computer interface issues (7). The remaining 60 participants were included in the ERP analyses.

### Materials

#### Stimuli

##### Naturalness categorization

Four hundred and eighteen scenic images were crowd-sourced via lab Facebook page and lab contacts. Instructions for submission of photos were as follows:

“The Multisensory Cognition Lab at USU is running a study on environmental perception, and is looking for participants to contribute stimuli to this study. If you would like to contribute some of your own photos, please send us your best photographs of different types of scenes – we are particularly looking for photos of a wide variety of natural settings and separate photos of manmade settings. Photos should be in color and should not contain people. They should not be easily recognizable landmarks that are commonly known to the general population. If we choose to use your photos in our experiment, they will first be rated by some of our participants and will then be viewed by other participants while they complete various cognitive tasks. They will not know you took the photos. You will not receive any compensation for or feedback about your photos. Please email childcognitioncenter@gmail.com with up to 10 photos if you would like to participate. Thanks!”

To determine categories of natural vs. built indicated by explicit behavioral choice, participants in the categorization sample were asked to categorize each of the 418 images as “natural” or “built” by placing their index fingers on the “q” and “p” keys on a standard keyboard and pressing “q” for “natural” and “p” for “built.” The images were presented on a 19″ LCD monitor (aspect ratio 4:3) in randomized order across participants, in a self-paced task, via Eprime. Images rated as “natural” by 60% of these participants or more were considered “natural” stimuli, and those categorized as “natural” by 40% or less were considered “built” stimuli. This resulted in 227 natural and 162 built images submitted.

##### Perceived restorativeness ratings

The 418 images were randomly assigned to 8 subsets of images so that sets were a manageable size to be rated in one sitting by participants. Each subset was rated by 6–9 participants on the PRS-short items. This rating procedure and sample sizes of the rating groups were designed to be consistent with that of Berto (2005). Images within a subset were presented in randomized order across participants. Participants were instructed to view each image, read each statement carefully, and assess whether the statement applied to how they would experience the place depicted in the image. Images remained on the screen while the participants recorded their responses on a response sheet; this involved an 11-point (0–10) rating scale beside each statement, through which they would select from ‘0’ indicating ‘not at all’ to 10 indicating ‘very much.’ After answering all items for one image, the participant pressed the spacebar to proceed to the next. The average restorativeness score across participants for each image was calculated.

**FIGURE 1:**
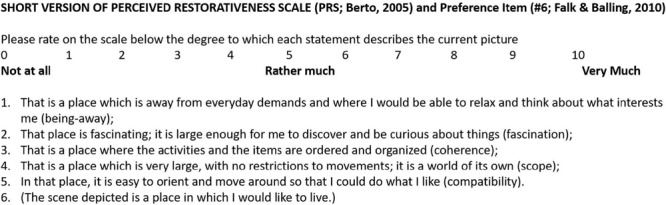
Participants in the rating sample were instructed to rate each picture on the five PRS-short items (Berto, 2005) and the additional preference item (item 6 above; Roe et al., 2013).

One further item of scene “preference” was included to be rated on the same 11-point (0–10) Likert scale as the PRS-short. The preference item, “The scene depicted is a place in which I would like to live,” was adapted from Falk and Balling’s (2010) investigation of landscape preference. See Figure 1 for the PRS short version including the additional preference item by Falk and Balling (2010).

#### EEG Data Acquisition

The EEG data were acquired using the EEG Emotiv headset and TestBench software with 14 channels (AF3, F7, F3, FC5, T7, P7, O1, O2, P8, T8, FC6, F4, F8, and AF4) using Common Mode Sense active electrode and Driven Right leg passive electrode (CMS/DRL) references at P3/P4; left/right mastoid process alternative. Samples were collected at a rate of 128 per second (2,048 Hz internal) and scalp impedance were below 10 kΩ at recording onset. Data were preprocessed in EEGLab (Delorme and Makeig, 2004). Data were filtered (bandpass: 0.1–59 Hz), re-referenced to average scalp reference, and trials containing artifacts were removed algorithmically based on abnormal trend and improbable data (Delorme and Makeig, 2004). Independent component analysis was used to reject stereotypical eye blinking and jaw-related muscle clenching. Each trial length spanned 1,200 ms, where the first 200 ms served as baseline for the 1,000 ms post-event epoch.

### Passive Oddball Viewing Task Procedure

**FIGURE 2:**
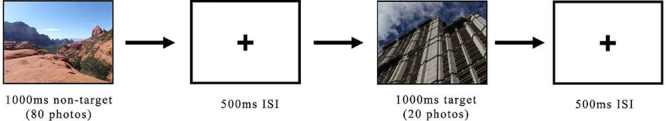
Images were shown for 1,000 ms followed by a 500 ms fixed-crossed inter-stimulus interval. In between blocks, a 45 s rest occurred.

Participants were seated in a chair 24 inches from the monitor. The experimenter placed the EEG cap on the participant’s head and ensured that all electrode sites showed an impedance of less than 10 kΩ before starting data recording. Participants completed a passive oddball task with instructions as follows: “You will be seeing a series of pictures. View the images as they appear.” After the instructions, the EEG headset was equipped for recording from participants. Participants were shown two blocks consisting of various images. Half of the participants first viewed the natural-non-target block, in which 80 natural and 20 built images were presented in random order. In the built-non-target block, image category frequencies were reversed. For each participant, the experimental software was programmed to select images randomly for non-target and target roles in the respective blocks from all images in the “nature” and “built” categories as determined by the results of the categorization task described above. Thus, two participants would not necessarily see all of the same images by the end of the experiment. The second half of participants received these blocks in the opposite order. Images were shown for 1,000 ms followed by a 500 ms fixed-cross inter-stimulus interval (Figure 2). In between blocks, a 45 s rest occurred.

### ERP Analysis

For all ERP analysis the grand mean was taken across standard and target epochs. The p3 analysis window was defined as 200–400 ms (Rozenkrants and Polich, 2008). P300 amplitude was measured at AF3, F7, F3, FC5, T7, P7, O1, O2, P8, T8, FC6, F4, F8, and AF4 from base to peak. P3 was calculated by taking the percent change of the p3 window relative to the ISI. This was then aggregated to calculate the grand means across channels. Mean peak amplitudes were used to derive the p3 across all participants for each condition. A 3-way repeated measures ANOVA for Environment (natural and built) × Stimulus (target and non-target) × Channel (14 channels) was conducted on p3 mean amplitude. A planned comparison excluding the Stimulus factor was also conducted in order to further isolate effects specific to Environment in the different Stimulus conditions. The LPP was defined as the mean amplitude at 550–930 ms after the target stimulus was presented (MacNamara et al., 2011). An initial 3-way repeated measures ANOVA (rmANOVA) for Environment (natural and built) × Stimulus (target and non-target) × Channel (14 channels) was also conducted on LPP mean amplitude. Achieved power (1 − β) was 1.00. Statistical analyses were performed in JASP (JASP Team, 2016). Analysis of variance (ANOVA) methods were used for group level comparisons. To correct for sphericity violations, Greenhouse–Geisser was applied for all ANOVA comparisons.

## Results

### Behavioral Results: Restorativeness Ratings and Categorical Preferences

Perceived restorativeness averages of the two environmental categories were compared using an independent samples *t*-test. The natural images (*M* = 6.48, *SD* = 0.94) were rated as more restorative than the built images [*M* = 5.64, *SD* = 1.12; *t*(307.675) = 7.732, *p* < 0.001, *d* = 0.88]. Natural images were expectedly rated higher on fascination [*t*(312.204) = 8.599, *p* < 0.001, *d* = 0.97], being away [*t*(305.266) = 10.844, *p* < 0.001, *d* = 1.24], extent [*t*(316.204) = 16.863, *p* < 0.001, *d* = 1.89], and compatibility [*t*(304.169) = 8.637, *p* < 0.001, *d* = 0.99]. Notably, natural images were unexpectedly rated lower than built on coherence [*t*(306.882) = −15.634, *p* < 0.001, *d* = 1.78]. The assumption of equal variances was not met for the comparisons of overall restorativeness, being away, fascination, extent, and compatibility. Thus, test statistics accounting for this violation have been reported; however, this assumption was met for the coherence comparison, and values for that test are thus unadjusted. For descriptive statistics by category for each restorativeness subcomponent, see Table 1.

**TABLE 1:** Mean ratings by restoration subcomponent and environmental category.

**Subcomponent**	**Category**	**Mean**	***SD***
Fascination	Nature	7.33	1.29
	Built	6.07	1.51
Coherence	Nature	3.7	1.33
	Built	6.03	1.59
Extent	Nature	7.36	1.22
	Built	5.05	1.41
Being away	Nature	7.43	1.45
	Built	5.61	1.75
Compatibility	Nature	6.56	1.11
	Built	5.45	1.34
Preference	Nature	5.48	1.72
	Built	4.74	2.03

**Preference is not considered a subcomponent of attention restoration.**

A significant difference for preference between natural (*M* = 5.48, *SD* = 1.72) and built (*M* = 4.74, *SD* = 2.03) images was also revealed [*t*(309.599) = 3.755, *p* < 0.001, *d* = 0.43]. The assumption of equal variances was also not met for this comparison, with built images having significantly greater variance in preference than natural images (*F-max* = 1.39, *p* < 0.05), so the adjusted statistics are reported above.

### ERP Results

#### p3 Results

After conducting the analysis along p3 mean amplitude, we found no significant interaction effects between Environment × Stimulus × Channel. A significant main effect for Channel was found [*F*(2.462,145.25) = 162.37; *p* < 0.001; $\eta_{\text{p}}^{2}$ = 0.733, 1−β = 0.99], but no significant differences were found for Environment or Stimulus. A planned comparison excluding the Stimulus factor was also conducted in order to further isolate effects specific to Environment in the different Stimulus conditions, but no effect of Environment was found. Thus, no oddball effect of the 80/20% categorical frequency presentation scheme on attentional resources was shown. We failed to see a significant oddball effect for p3 [*F*(1,59) = 0.135; *p* = 0.715].

#### LPP Results

**FIGURE 3:**
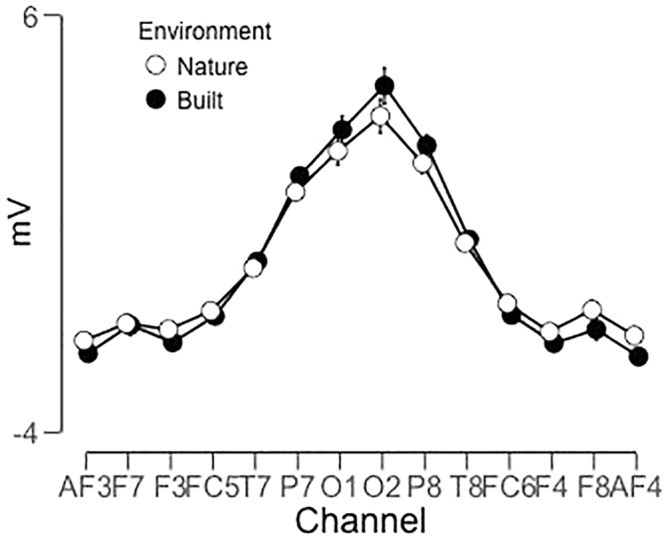
Environment × Channel interaction showing difference in polarity between posterior and frontal channels [*F*(4.36,257.34) = 4.36, *p* < 0.01, $\eta_{\text{p}}^{2}$ = 0.07].

**FIGURE 4:**
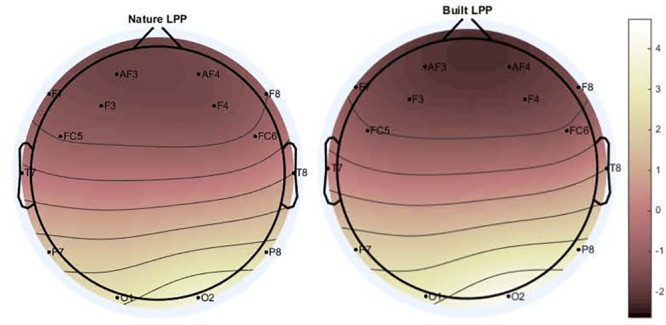
Main effect of Environment indicates natural stimuli elicit lesser LPP mean amplitude than built stimuli across all channels [*F*(1.97,116.25) = 50.845; *p* < 0.001; $\eta_{\text{p}}^{2}$ = 0.463]. Differences in frontal and posterior channel polarity were resolved by dividing subsequent analyses into frontal channels only (AF3, F3, F7, FC5, FC6, F4, F8, and AF4) and posterior channels only (T7, P7, O1, O2, P8, and T8).

**FIGURE 5:**
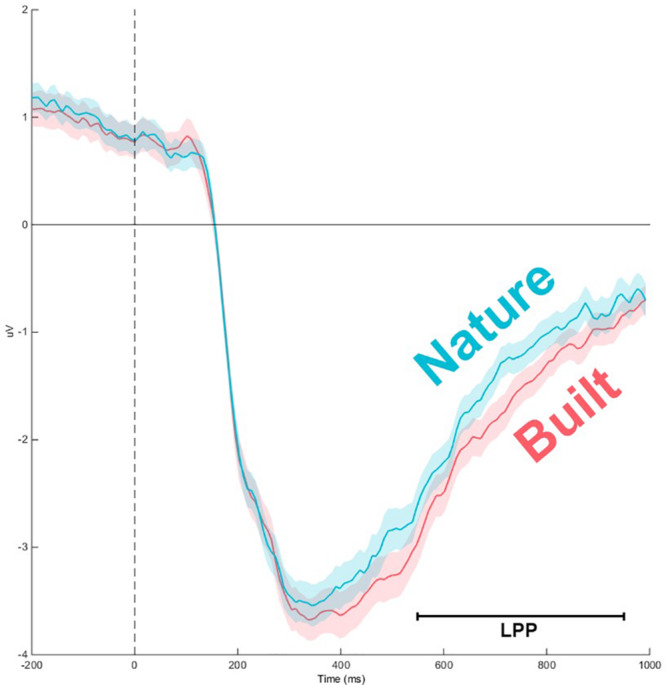
Event-related potentials time course for frontal channels (averaged). Main effect of Environment indicates natural stimuli to elicit lesser LPP mean amplitude than built stimuli in frontal channels [*F*(1,59) = 11.12; *p* < 0.005; $\eta_{\text{p}}^{2}$ = 0.16]. Error shading represents 95% confidence interval.

A significant main effect for Channel along LPP mean amplitude was revealed [*F*(1.97,116.25) = 50.845; *p* < 0.001; $\eta_{\text{p}}^{2}$ = 0.463] as well as a significant interaction for Environment × Channel [*F*(4.36,257.34) = 4.36, *p* < 0.01, $\eta_{\text{p}}^{2}$ = 0.07], which indicated a difference in polarity between frontal channels and posterior channels (Figures 3, 4). Two separate 3-way rmANOVA were conducted on these two areas to compensate for polarity differences, and *p*-values were subsequently Bonferroni corrected. In frontal channels, a significant main effect for Environment was found [*F*(1,59) = 11.12; *p* < 0.005; $\eta_{\text{p}}^{2}$ = 0.16], indicating a greater LPP mean amplitude for built environments than natural environments [*t*(59) = 3.34; *p* < 0.005]. A Bayesian rmANOVA was also conducted, indicating strong support in favor of built environments eliciting greater LPP activity than natural environments (BF_10_ = 28.08; posterior probability: 96.56%; Figure 5). In posterior channels, a significant main effect for Environment was also found [*F*(1,59) = 11.12; *p* < 0.005; $\eta_{\text{p}}^{2}$ = 0.16], indicating a greater LPP mean amplitude for built environments than natural environments [*t*(59) = 3.34; *p* < 0.005]. A Bayesian rmANOVA was also conducted, indicating support for built environments eliciting greater LPP activity than natural environments (BF_10_ = 2.32; posterior probability: 69.88%).

## Discussion

This study was designed to investigate, with ERP methodology and a passive oddball paradigm, whether viewing natural versus built environments correlates with distinct electrophysiological patterns. Unlike previous, primarily behavioral investigations, this study utilized temporally sensitive electrophysiological measures to examine potential differences in neural responses while viewing natural versus built environments. Our findings indicate lesser LPP amplitude and faster LPP recovery time when the non-target stimuli were those of natural environments versus built environments. Data suggest that participants perceived natural scenes as more pleasant than built scenes. Results also suggest that, in addition to explicit behavioral categorization, there may be an implicit categorization of natural and built environments that can be revealed through distinct physiological signatures. The current results leave open the question of whether stimuli were implicitly categorized as a function of naturalness, restorativeness, or both. Future studies should experimentally distinguish between these stimulus constructs.

Subjective ratings revealed greater perceived restorative potential for natural images than for built images. This finding is consistent with previous empirical studies of environmental imagery (Berto, 2005; Berman et al., 2008). While we did not collect self-report ratings of image emotional valence, we did find that preference was greater for natural environments than built. This suggests more positive association with natural images compared to built images when behavioral preference is explicitly measured. Given that previous research has found restorative environments to also be more explicitly preferred (Berto, 2005; Aspinall et al., 2013), it is suspected that the greater perceived restorative potential of the nature images used in this study underlies participants’ demonstrated greater implicit preference for them. Further, some research has begun to explore the basic visual characteristics of natural scenery that may underlie the cognitive and emotional benefits of this scenery. For example, similar to Aspinall et al. (2013) findings of greater alpha wave activity when viewing natural scenery, Hägerhäll et al. (2015) found that viewing statistical fractals – the type of repeating visual patterns characteristic of natural scenery – correlated with greater alpha wave activity compared to exact fractal patterns – those characteristic of human architecture. As alpha activity is associated with a wakeful relaxed state and attentiveness, it may be that the same visual characteristics facilitating attention restoration may implicitly cue scene category.

Convergent, implicit electrophysiological evidence of this association derives from the lesser LPP activation found while viewing greater frequencies of natural compared with built images. In previous research, lesser LPP amplitude for valent stimuli has been indicative of greater perceived pleasantness (Hajcak and Nieuwenhuis, 2006; Hajcak and Olvet, 2008; MacNamara et al., 2011) as well as implicit and explicit categorization processes (Ito and Cacioppo, 2000). Since viewing natural scenes correlated with lesser LPP amplitude when compared to built scenes in the current study, it is plausible that natural scenes are more emotionally valent, and particularly more pleasant, than built scenes, even when viewed passively with no explicit instructions for categorization.

Late positive potential differences in response to viewing natural versus built stimuli were more apparent over frontal sensor sites than parietal or occipital sites (which showed significance, but with weaker consistency as determined by Bayesian analyses). Liu et al. (2012) LPP-BOLD coupling revealed between-category (pleasant, neutral, and unpleasant) differences in LPP amplitude, BOLD activity, and recruited neural substrates. These substrates’ level of contribution to LPP modulation was also valence-specific. In the context of previous findings, we take the significant difference in LPP amplitude to indicate potential differences in perceived valence between natural and built scenes. However, LPP’s being evoked by mere arousal, rather than also being moderated by valence, is debated, as cognitive neuroscientific conclusions are inherently prone to reverse inference (e.g., Hajcak and Nieuwenhuis, 2006; Poldrack, 2011). In fact, research has shown the effects of arousal and valence on LPP to be difficult to tease apart, though some differences in return to baseline have been found between high-arousing unpleasant images and pleasant images (O’hare et al., 2017).

A recent review by Hajcak and Foti (2020) concluded that even more so than arousal, LPP may likely be modulated specifically by motivational significance. This significance is that which facilitates approach and avoidance responses to stimuli, whether task relevant or not. Thus, it is plausible that the LPP from the current EEG sample correlates with such responses to both natural and built stimuli. Further, Gable and Adams (2013) found that paradigms with a long stimulus duration (e.g., 1,000 ms) elicit a more protracted p3, similar to the LPP elicited in an emotional viewing task, while oddball paradigms with a short stimulus duration (e.g., 200 ms) elicited a more typical p3. That stimuli in the current study were displayed for 1,000 ms, but the p3 analysis window was restricted to 200–400 ms, could explain the lack of p3 differences found between conditions.

This lack of a significant oddball effect in the current study, though surprising, may also be due to the use of a passive task. Given that the standard and target images were determined at the category level, perhaps an *active* oddball task, in which participants received instructions to specifically search for, and respond to, various exemplars of the low frequency target category, using the current stimuli, may have shown p3 differences, and thus an oddball effect—though this remains to be tested. In combination with the passive nature of the task, another limiting factor with respect to examining the p3 may have been within-category stimulus diversity: the large number of unique images within each category. The p3 component is traditionally studied using one exemplar of a category in each of the target and non-target roles (Polich, 2003; Rozenkrants and Polich, 2008). A design incorporating within-category stimulus homogeneity may more directly elicit a p3 component. Another limitation is that, given our stimulus categorization thresholds (>60% = natural, <40% = built), the lack of a significant p3 finding could also be due to the inclusion of environmentally heterogeneous images. For example, a natural image that was categorized as “natural” by 61% of the categorization sample may still contain a substantial number of built environment features. Operationalizing the natural and built categories at the chosen thresholds was necessary, though, to ensure sufficiently large stimulus sets in the current study. Furthermore, within-environment stimulus diversity was necessary in order to draw category-level conclusions for environment. Previous behavioral studies of natural versus built environments have also employed diverse stimulus sets (Berto, 2005; Berman et al., 2008; Roe et al., 2013; Pasini et al., 2014), prompting the neural comparison design reported herein. Questions concerning potential methodological limitations, however, should be addressed in future research.

### Future Directions

The current results leave open the question of whether stimuli were implicitly categorized as a function of naturalness, restorativeness, both, or possible correlating low-level visual features (Kotabe et al., 2017); the variables correlate in the current design. Natural stimuli within this study were rated higher in four of the five components of ART, indicating natural environments in this study to be more restorative than built environments. Since the design of this study relies on a passive task measuring implicit categorization, it is possible that components of ART requiring greater implicit (bottom-up) processing are most critical in determining categorical differences in restoration. An example of one such component could be *fascination*, or how an environment captures exogenous attention. In the current study, though, we cannot separate out the different components of ART for each given stimulus in order to accurately assess whether the differences are truly due to restoration as opposed to the ‘naturalness’ of environmental categories (e.g., Scopelliti et al., 2019). Image statistics and the time course of visually processing natural vs. non-natural scenes may also drive the implicit categorization of these two environment types. The fractal structure in natural scenes may be more quickly and easily processed than the straighter structure in man-made scenes, and contextual cues to category may derive from quickly established global action (affordance) or function related properties of environments—rather than merely segmented, local objects and parts (e.g., Torralba and Oliva, 2003; Green and Hummel, 2004; Greene and Oliva, 2006, 2009; Oliva and Torralba, 2006, 2007). Future research should further measure and characterize the statistical properties in scenes to help elucidate mechanisms underlying the current findings (e.g., Geisler, 2008). Future research should also further address neural correlates with natural versus built environments using stimuli presented in modalities other than vision. Previous studies suggest that organisms may judge soundscape environments as pleasant based on perceived indicators of safety – though in contrast to the visual processing discussed above, this type of auditory processing is subcortical (e.g., Andringa and Lanser, 2013; van den Bosch et al., 2018). It would be interesting if across modality, findings converged to show that organisms more rapidly and easily categorize natural environments as safe and therefore also as more pleasant.

Although participant ratings of the stimuli used in this study revealed natural scenes to be more restorative overall than built scenes, natural scenes were surprisingly lower than built scenes along the ‘coherence’ component. To clarify this result, replications using these stimuli should collect negative- and positive-specific valence ratings. Also, further ratings using the PRS-short should be collected to ensure that the direction of the coherence difference reported herein is not spurious. It is also possible that ‘coherence’ was interpreted by participants differently than it has been defined within ART; perhaps, for example, participants rated coherence lower in natural scenes since unlike built environments, they are not designed for some human-driven purpose. Finally, future research should explore correlations between specific ART components and positive and negative valence, as well as performance on directed attention tasks. It may be that some ART components - expectedly, being away and compatibility – may contribute more to positive valence of a scene while others, such as fascination, extent, and coherence, are more strongly related to replenishment of attentional resources as suggested by previous ART research. Although the work presented here is only an initial exploration, understanding the role of positive experience within attention restoration—including from a neural perspective – is a ripe area for future investigation. A neural methodological perspective will help us understand more completely the various health benefits humans can accrue from exposure to natural environments.

## Data Availability Statement

The raw data supporting the conclusions of this article will be made available by the authors, without undue reservation, to any qualified researcher.

## Ethics Statement

The studies involving human participants were reviewed and approved by Utah State University IRB. The patients/participants provided their written informed consent to participate in this study.

## Author Contributions

SM, NW, AP, AH, JC, TL, and KJ wrote the manuscript. SM, NW, AP, AH, TL, and JC collected the data. SM, AH, NW, AP, and JC performed the data analysis. SM, NW, and KJ conceptualized the study.

## Conflict of Interest

The authors declare that the research was conducted in the absence of any commercial or financial relationships that could be construed as a potential conflict of interest.
